# Evaluation of Dizziness in the Emergency Department: Prevalence and Diagnostic Utility of Clinical Scales for Functional Vertigo

**DOI:** 10.5811/westjem.47389

**Published:** 2025-12-31

**Authors:** Melis Dorter, Yusuf Koksal, Can Aktas

**Affiliations:** * Primary Health Care Corporation, Doha, Qatar; † Koç University Hospital, Department of Emergency Medicine, Istanbul, Türkiye

## Abstract

**Introduction:**

Functional vertigo is commonly missed in the emergency department (ED) and often misdiagnosed as other peripheral vestibular disorders. It is strongly associated with anxiety and depression, yet standardized diagnostic criteria are lacking in the ED setting, leading to unnecessary tests and misdiagnosis. We aimed to assess the diagnostic accuracy of the Vertigo Symptom Scale - Short Form - Autonomic (VSS-SF-A) and the Hospital Anxiety and Depression Scale – Anxiety (HADS-A) and – Depression (HADS-D) for distinguishing functional vertigo from other peripheral vertigos in the ED and to determine its prevalence.

**Methods:**

This was a prospective, cross-sectional, observational studey of adult patients of a tertiary-care ED with dizziness.. We included patients who received an initial peripheral vertigo diagnosis from attending emergency physicians. Blinded otolaryngologists (ENT) verified all final diagnoses through standardized evaluation methods performed on the same day as the ED visit. We excluded patients with central, metabolic, cardiovascular conditions. Study participants received thorough vestibular evaluations while a separate physician, also blinded to diagnostic outcomes, administered the VSS and HADS tests, which typically require 15–20 minutes to complete. The final ENT evaluation served as the criterion reference for the diagnosis of functional vertigo. We evaluated the diagnostic accuracy of the scales through receiver operating characteristic (ROC) analysis.

**Results:**

During the study period, 694 patients presented to the ED with dizziness-related complaints, of whom 69 (9.9%) met the inclusion criteria and were enrolled in the study. Of 69 patients initially diagnosed with peripheral vertigo in the ED, ENT specialists confirmed functional vertigo in 25 (36.2%) and peripheral vertigo in 44 (63.8%). Functional vertigo patients were significantly younger (43.4 ± 16.9 vs 60.1 ± 14.9 years of age, P < .001). In patients with functional vertigo, the mean VSS-SF-A, HADS-A, and HADS-D scores were 9.04, 9.28, and 7.52, respectively, compared to 3.80, 4.18, and 2.91 in peripheral vertigo cases. Conversely, the VSS-SF subscale—Vestibular-Balance (VSS-SF-V)—scores were higher in peripheral vertigo patients (13.05 vs 6.56), all P < .001. The ROC analysis showed that VSS-SF-A (cutoff ≥ 8, area under the curve [AUC] 0.85, 95% CI, 0.76–0.94) had the highest accuracy for diagnosing functional vertigo, with a sensitivity of 72% and specificity of 84.1%, followed by the HADS-A (cutoff ≥ 8, AUC = 0.81, 95% CI, 0.70–0.91), which had a sensitivity of 68% and specificity of 88.6%, while HADS-D (cutoff ≥ 4, AUC = 0.80 95% CI, 0.60–0.90) showed 76% sensitivity and 75% specificity.

**Conclusion:**

Functional vertigo is an underdiagnosed condition that produces dizziness in patients. The Vertigo Symptom Scale and Hospital Anxiety and Depression Scale show promise for enhancing early diagnosis while reducing unnecessary imaging and improving patient care. Future research is needed to confirm these findings through larger multicenter cohorts.

## INTRODUCTION

Tertiary-referral dizziness units diagnose functional vertigo in approximately 19.5% of adult patients presenting with dizziness, which is the second most common diagnosis after benign paroxysmal positional vertigo.^1^ Functionl vertigo, also known as psychogenic-somatoform vertigo or functional dizziness, describes dizziness that stems from psychological origins rather than structural problems. This condition produces head-turning and body-rocking sensations, which distinguish it from the typical spinning sensation found in somatic vertigo.^2^,^3^ Many patients present to the emergency department (ED) with vertigo and dizziness as their main symptoms. According to population-based studies these conditions affect 17–30% of the population and account for 3.5–11% of total ED admissions. The number of patients visiting the ED for dizziness has risen by approximately 50% during recent years.^4^–^6^

Previous studies have reported that functional vertigo may represent 30–50% of all dizziness cases, thus making it one of the leading causes of dizziness in outpatient and hospital environments.^7^,^8^ Despite the high prevalence of vertigo cases in ED settings, distinguishing peripheral vestibular disorders from functional vertigo is still challenging. Peripheral vestibular disorders such as benign paroxysmal positional vertigo, vestibular neuritis, and Ménière’s disease have well-defined clinical and diagnostic criteria. But there are no standardized approaches for diagnosing functional vertigo in the ED.^9^ Often, the diagnosis is made by ruling out all other possible causes and relies on the physician’s clinical judgment.^10^ In this study we aimed to fill this knowledge gap through an evaluation of the Vertigo Symptom Scale (VSS) and the Hospital Anxiety and Depression Scale (HADS) for diagnosis of functional vertigo in ED patients.

Misdiagnosis of vertigo subtypes, particularly failure to identify central causes, can lead to unnecessary imaging, delays in appropriate treatment, and increased healthcare costs.^11^,^12^ The close relationship between functional vertigo and anxiety, depression, and psychosomatic disorders indicates a multifaceted relationship between vestibular and psychological factors.^13^ Jang et al discovered a substantial link between depression and dizziness, which indicates that mood disorders frequently produce dizziness symptoms because they affect both the vestibular system and related brain areas.^14^ Anxiety stands as a primary symptom that patients with functional vertigo commonly experience.^1^,^15^ The HADS is a validated psychometric tool used to assess anxiety and depression in hospital settings. It has two subscales: HADS-Anxiety (HADS-A) and HADS-Depression (HADS-D), each with seven items scored on a 0–3 scale, with total scores ranging from 0 to 21. Higher scores on the HADS-A indicate increased anxiety symptoms, while higher scores on HADS-D suggest clinically significant depressive symptoms. This scale is widely used in both clinical and research settings for screening and assessing the severity of psychiatric comorbidities in various medical conditions.^16^

**Population Health Research Capsule**

What do we already know about this issue?

*Functional vertigo is often misdiagnosed in the emergency department (ED), leading to unnecessary tests and delayed management*.

What was the research question?

*Can the Vertigo Symptom Scale (VSS) and Hospital Anxiety and Depression Scale differentiate functional from peripheral vertigo in ED patients?*

What was the major finding of the study?

*A score ≥ 8 on the VSS-Short Form-Autonomic-Anxiety diagnosed functional vertigo with 72% sensitivity and 84% specificity (AUC = 0.85, P < .001)*.

How does this improve population health?

*Early detection of functional vertigo may reduce imaging, lower healthcare costs, and improve patient outcomes in ED care*.

The VSS represents a validated assessment instrument that evaluates dizziness and vertigo symptom intensity. The VSS-Short Form Vestibular (VSS-SF-V) and VSS-Short Form Autonomic (VSS-SF-A) subscales contain eight vestibular- and balance-related items and seven autonomic- and anxiety-related items. The scoring system uses a 0–4 scale for each item to determine overall symptom severity. The VSS-SF-V subscale assesses vestibular symptoms including vertigo and imbalance and motion-induced dizziness to help identify peripheral vestibular disorders. The VSS-SF-A subscale evaluates autonomic symptoms including sweating. nausea and palpitations, which commonly occur in functional vertigo.^17^

We hypothesized that implementing psychometric scales during standard ED assessments would boost the accuracy of functional vertigo detection, while decreasing the number of incorrect diagnoses and unneeded medical procedures. The current medical literature lacks an objective method to diagnose functional vertigo. We sought to evaluate the VSS and HADS along with their subscales as potential diagnostic tools for ED patients with dizziness. The implementation of psychometric assessments in clinical practice will lead to better diagnostic precision and more efficient patient care and improved treatment results. Our secondary aim was to establish the real prevalence of functional vertigo among patients presenting to the ED who were initially diagnosed with peripheral vertigo.

## METHODS

### Study Objective

We aimed to assess the utility of the HADS and the VSS in diagnosing functional vertigo in patients presenting with dizziness to the ED after ruling out all central causes. The original scales used were validated and found to reliable and trustworthy in the Turkish language.^13^,^18^ Our secondary objective was to establish the true prevalence of functional vertigo among patients who were initially thought to have peripheral vertigo when they arrived at the ED.

### Study Design

We conducted this prospective, cross-sectional, observational study between January 2023–January 2024 in the Department of Emergency Medicine at Koç University Hospital, a tertiary-care center in Istanbul, Türkiye, treating approximately 43,000 patients annually in its ED. We evaluated the occurrence of functional vertigo among dizziness patients while assessing the VSS and HADS scales as diagnostic tools. A separate physician, blinded to the diagnostic outcomes, administered the VSS and HADS tests. These self-reported questionaries are brief and straightforward, with a total completion time of approximately 15–20 minutes, depending on the patient’s condition and reading speed. Patients underwent otolaryngological (ENT) consultations and comprehensive vestibular assessments performed on the same day as their ED visit. The final clinical opinion of the ENT specialist served as the criterion reference for the diagnosis of functional vertigo.

The Koç University Ethics Board approved the study (approval number: 2023.276, IRB1.090), while all participants gave their written consent to participate. The study follows the Strengthening the Reporting of Observational Studies in Epidemiology (STROBE) and Standards for Reporting Diagnostic Accuracy Studies (STARD) guidelines for reporting findings.^19^,^20^ There were no missing data for primary variables.

### Study Population and Patient Selection

Between January 2023–January 2024, a total of 694 patients presented to the Koç University Hospital ED with dizziness-related complaints.

### Inclusion Criteria

Patients were eligible for inclusion if they were ≥ 18 years of age, presented to the ED with acute or subacute dizziness, and were initially presumed to have peripheral vertigo by the attending emergency physician. Additionally, patients were required to have undergone an ENT consultation for vestibular assessment and received a definitive diagnosis based on ENT evaluation and vestibular testing.

### Exclusion Criteria

Patients were excluded if their dizziness was attributed to a confirmed central pathology such as stroke, vestibular migraine, or multiple sclerosis. Other exclusion criteria included dizziness due to metabolic or cardiovascular causes, pregnancy, and lack of ENT consultation—typically due to the unavailability of ENT physicians during night shifts or weekends.

**Figure 1:**
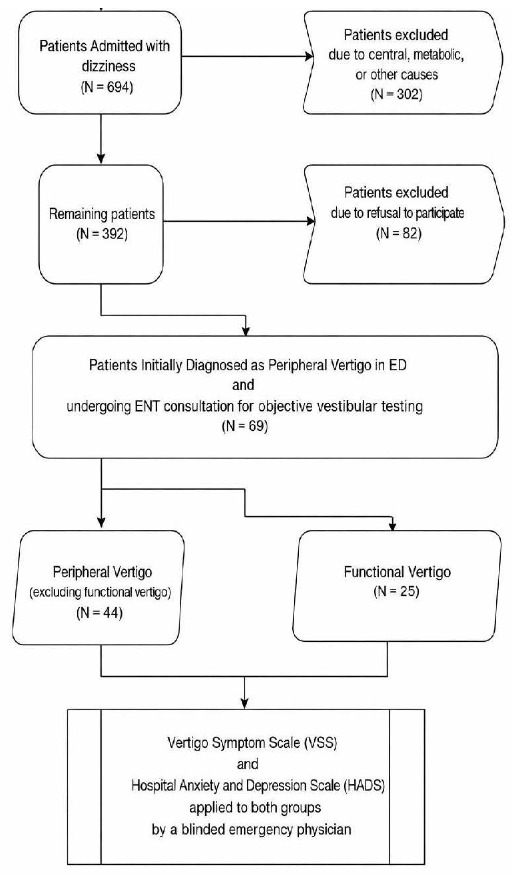
Patient flowchart in a study of dizziness-related emergency department visits. *ED*, emergency department; *ENT*, Ear-nose-throat; *VSS*, Vertigo Symptom Scale; *HADS*, Hospital Anxiety and Depression Scale.

The study included 69 patients who received a complete ENT evaluation. Forty-four patients were diagnosed with peripheral vertigo conditions such as benign paroxysmal positional vertigo, vestibular neuritis, and Ménière’s disease while 25 patients received a final diagnosis of functional vertigo because they showed no structural vestibular abnormalities despite ongoing dizziness symptoms (see Figure 1).

### Emergency Department and ENT Specialist Evaluation

The ED evaluation started by eliminating central causes of vertigo through neurological examination, review of cranial imaging (when available), and clinical evaluation using the timing and triggers approach as outlined in recent emergency medicine guidelines during the first.^12^ The Head-Impulse, Nystagmus, Test of Skew assessment was performed only in patients with continuous vertigo and no obvious auditory symptoms, following the timing-and-triggers approach to differentiate central from peripheral causes. The assessment included orthostatic blood pressure tests to detect autonomic dysfunction.

The evaluation process for patients with suspected peripheral vertigo involved a complete vestibular examination by ENT specialists if the ENT clinic was available. The ENT specialists were blinded to the patient’s condition. This assessment included the following:

- Dix-Hallpike maneuver to identify benign paroxysmal positional vertigo
- Audiometric testing to assess co-existing cochlear involvement
- Videonystagmography to evaluate spontaneous and positional nystagmus
- Caloric testing (bithermal irrigation) to assess unilateral vestibular hypofunction
- Posturography with the Romberg test to evaluate postural stability.

The diagnosis of functional vertigo was made for patients who had no structural vestibular deficits but continued to experience dizziness symptoms that did not match their test results. The final classification for peripheral vertigo was made according to a definitive objective diagnosis, whereas the classification of functional vertigo was made by clinical judgment, the exclusion of all other vestibular disorders, negative findings on previously mentioned examinations, and symptom patterns suggestive of functional dizziness.

### Assessment of Anxiety and Depression

The VSS and the HADS were used to evaluate the potential psychological factors in functional dizziness for all participants. We used the VSS to evaluate vestibular and autonomic symptoms and the HADS to evaluate clinically significant anxiety and depressive symptoms. The assessments were administered by an emergency physician who was not aware of the patient’s final diagnosis to minimize bias.

### Data Collection and Statistical Analysis

The research design for this study involved a prospective, cross-sectional, observational approach to examine adult patients who visited a tertiary-care ED with dizziness symptoms The analysis used patient data obtained from electronic health records that contained demographic information together with clinical characteristics and vestibular test results. Patients who provided consent and had a final diagnosis confirmed by ENT and objective tests, completed the questionnaires. To minimize bias, a blinded emergency physician who was not involved in the initial assessment independently analyzed the VSS and HADS scores without knowledge of the patients’ final diagnoses. All statistical analyses were performed using SPSS Statistics v20.0 (IBM Corporation, Armonk, NY) and Jamovi v2.4.11 (The Jamovi Project, Sydney, Australia). The normality of data distribution was assessed using the Shapiro-Wilk and Kolmogorov-Smirnov tests. We presented data as mean ± standard deviation for continuous variables and percentages for categorical variables. We used the Student *t*-test together with the chi-square test and Pearson correlation analysis for between-group comparisons, and we used receiver operating characteristic (ROC) curve analysis to assess the diagnostic precision of VSS and HADS scores for distinguishing functional from peripheral vertigo. A *P*-value < .05 determined as statistical significance.

### Power Analysis

We conducted a G Power analysis to determine the statistical power of the study. The lowest expected Pearson correlation (*R*^2^ = .37) was applied using bivariate normal modeling, with an alpha error of *P* < .001 and a total sample size of 69; the study demonstrated a statistical power of 99%, indicating a strong likelihood of detecting true associations between anxiety, depression, and functional dizziness.

### Ethical Consideration

The research followed the principles of the Declaration of Helsinki together with the ethical rules of the institution. The Koç University Ethics Board granted ethical approval (2023.276 (IRB1.090). The participants gave their consent through written statements before data collection started, and researchers maintained the patients’ confidentiality throughout the entire study. The research data received protection through anonymization, and only authorized personnel had access to the data.

## RESULTS

The study included 69 patients after evaluations and ENT specialist consultations; 25 patients had functional vertigo, and 44 patients had peripheral vertigo (Figure 1). The study revealed that in our patient population the prevalence of functional vertigo occurred in 36% of cases, which may not be generalizable to other settings due to our single-center design.

The mean age of patients with functional vertigo was significantly younger (43.44 ± 16.85 years of age) compared to those with peripheral vertigo (60.14 ± 14.93, *P* < .001). The sex distribution was not significantly different between the two groups, with 60% females in the functional vertigo group and 54.5% females in the peripheral vertigo group (*P* = .66) (Table 1).

The VSS-Short Form-Total (VSS-SF-T) scores were comparable between both groups, suggesting no difference in the overall severity of vertigo symptoms (*P* = .66). However, scores on the VSS-Short Form-Autonomic (VSS-SF-A), HADS-Anxiety (HADS-A), and HADS-Depression (HADS-D) were significantly higher in the FV group (*P* < .001), whereas scores on the VSS-Short Form-Vestibular (VSS-SF-V) subscalewere significantly higher in the peripheral vertigo group (*P* < .001) (Table 1).

The Pearson correlation analyses are summarized in Table 2. The VSS-SF-A scores demonstrated strong correlations with both HADS-A (*r* = 0.695, *P* < .001) and HADS-D (*r* = 0.609, *P* < .001) scores, which showed higher scores on these assessments in patients diagnosed with functional vertigo.

**Figure 2:**
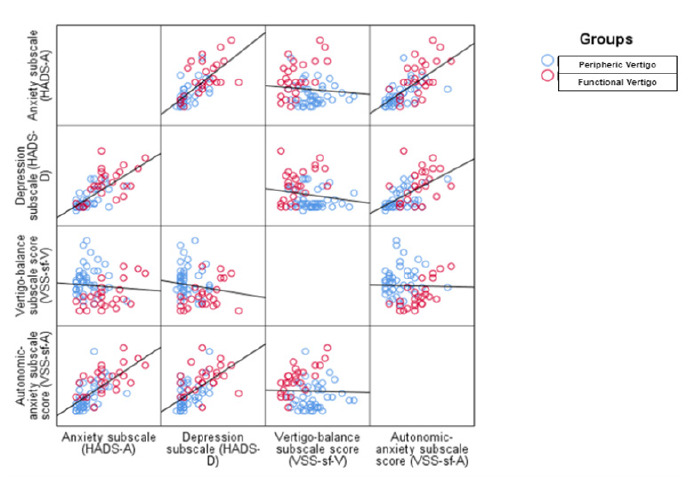
Matrix graph of patient distributions and correlations in a study of vertigo subtypes among emergency department patients. This figure provides a visual representation of the distribution of patients and the correlations among scores on the following scales: VSS-SF-A (Autonomic-Anxiety Subscale), VSS-SF-V (Vertigo-Balance Subscale), HADS-A (Anxiety Subscale), and HADS-D (Depression Subscale). Patient data are stratified by their final clinical classification as either functional vertigo (red) or peripheral vertigo (blue).The diagonal panels display individual variable distributions while the lower left panels show pairwise correlation plots between scales. This visual summary highlights how anxiety-related and autonomic symptoms are inter-related in different vertigo subtypes. *VSS-SF-V*, Vertigo-balance subscale score; *VSS-SF-A*, Autonomic-anxiety subscale score; *HADS-A*, Anxiety subscale of the Hospital Anxiety and Depression Scale; *HADS-D*, Depression subscale of the Hospital Anxiety and Depression Scale.

The VSS-SF-V scores showed an inverse relationship with VSS-SF-A, HADS-D and HADS-A scores. The pattern of high VSS-SF-V scores with low scores on other assessments was characteristic of peripheral vertigo. The patient distribution and inter-scale correlation matrix is shown in Figure 2, which shows that elevated VSS-SF-A, HADS-A, and HADS-D scores are more pronounced in functional vertigo (Figure 2).

**Figure 3:**
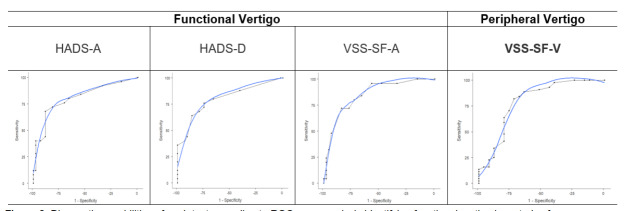
Diagnostic capabilities of each test according to ROC curve analysis identifying functional vertigo in a study of emergency department patients. This figure shows ROC curves for each diagnostic measure in differentiating functional vertigo from peripheral vertigo. The AUC values, optimal cut points, and associated sensitivity/specificity values are indicated for each measure. Such metrics guide clinicians in identifying the most predictive thresholds for each scale. *VSS-SF-V*, Vertigo-balance subscale score; *VSS-SF-A*, Autonomic-anxiety subscale score; *HADS-A*, Anxiety subscale of the Hospital Anxiety and Depression Scale; *HADS-D*, Depression subscale of the Hospital Anxiety and Depression Scale; *ROC*, receiver operating characteristic; *AUC*, area under the curve.

ROC curve analysis (Figure 3) for diagnostic accuracy showed the following:

- A HADS-A score threshold of ≥ 8 provided the highest diagnostic accuracy for functional vertigo, with a sensitivity of 68%, specificity of 88.64%, and an area under the curve (AUC) of 0.807.
- A HADS-D score threshold of ≥4 demonstrated optimal diagnostic values with a sensitivity of 74%, specificity of 75%, and an AUC of 0.804.
- A VSS-SF-A score threshold of ≥ 8 demonstrated optimal diagnostic accuracy, with a sensitivity of 72%, specificity of 84.09%, and an AUC of 0.847.
- For diagnosing other types of peripheral vertigo, a VSS-SF-V score threshold of ≥ 9 yielded a sensitivity of 81.82%, specificity of 72%, and an AUC of 0.795.

The results for positive and negative predictive values are summarized in Table 3. A ROC analysis showed that VSS-SF-A (cutoff ≥ 8, AUC = 0.85, 95% CI, 0.76–0.94) had the highest accuracy for diagnosing functional vertigo, with a sensitivity of 72% and specificity of 84.1%, followed by HADS-A (cutoff ≥ 8, AUC = 0.81, 95% CI, 0.70–0.91) had a sensitivity of 68% and specificity of 88.6%, while HADS-D (cutoff ≥ 4, AUC = 0.80, 95% CI, 0.60–0.90) showed 76% sensitivity and 75% specificity. The ROC curves in Figure 3 demonstrate that VSS-SF-A and HADS-A and HADS-D successfully differentiated functional from peripheral vertigo and VSS-SF-V successfully identified peripheral vertigo.

## DISCUSSION

### Prevalence and Overlooked Nature of Functional Vertigo

In the ED setting functional vertigo is often missed because this condition receives inadequate recognition and incorrect diagnoses. Our research showed that 36% of patients who received an initial peripheral vertigo diagnosis in the ED actually had functional vertigo without any detectable organic disease, which supports prior research results that non-organic dizziness cases represent 30–50% of all dizziness cases in clinical settings.^5^,^21^ Schmid et al (2011) reported that dizziness in many patients could not be fully explained by identifiable medical conditions, with most diagnostic tests failing to reveal pathological results.^22^ We found that functional vertigo remains a common condition that clinicians frequently mistake for peripheral vestibular dysfunction, underscoring the need for improved diagnostic methods in the ED.

### Diagnostic Accuracy of Psychometric Tools

Our research revealed that psychometric assessments are significantly associated with the differentiation of functional vertigo from peripheral vestibular disorders and may aid in diagnostic decision-making. The VSS-SF-A produced the best diagnostic results among the tested measures with an AUC of 0.847 when using a cutoff score of 8. The HADS-A and HADS-D tests showed diagnostic accuracy with AUC values of 0.807 and 0.804, respectively, when using cutoff scores of 8 and 4. The VSS-SF-V subscale scores demonstrated high diagnostic accuracy (cutoff ≥ 9, AUC = 0.795) in the peripheral vertigo group, which suggested that vestibular causes were the main issue for these patients—a finding supported by prior studies.^23^

Researchers have assessed the VSS for its ability to differentiate organic from functional vertigo. The “vertigo-related symptoms” subscale according to Tschan et al (2008) proved useful for distinguishing organic vertigo patients from healthy participants but the “somatic anxiety and autonomic arousal” subscale demonstrated only moderate success in differentiating functional vertigo. The results of that study match our findings; however, they used the full version of the scales in their research.^24^ In a study by Talaat et al in 2020 that was designed to validate the language of the Arabic version of the VSS, one of their secondary findings was that the Arabic version of the VSS-SF successfully distinguished patients with vestibular disorders from those with anxiety-related vertigo.^25^

Dyukova et al (2021) proposed that the VSS-SF could be used to predict the risk of developing functional vertigo in patients with high anxiety levels.^26^ The research conducted by Limburg et al with 72 participants demonstrated that functional vertigo patients exhibited elevated Beck Anxiety scores.^27^ Our research included patients with peripheral vertigo (benign paroxysmal positional vertigo included); the functional vertigo patients who received VSS and HADS assessments confirmed the need to evaluate for anxiety. Several studies have also explored the utility of HADS in patients experiencing dizziness. Chronic dizziness and vertigo are often associated with anxiety and depression.^28^ Piker et al (2015) examined the application of HADS for psychiatric symptom assessment in patients with dizziness. The authors determined that HADS successfully measured general psychological distress, but they found it inadequate for distinguishing between various vertigo subtypes. This was in contrast to the results of our study, which showed that HADS-A and HADS-D scores demonstrated better ability to distinguish functional vertigo from organic vertigo compared to their findings.^29^

A study by Tschan et al (2008) indicated that HADS could help in distinguishing between functional vertigo and organic vertigo, although with moderate effectiveness. They found that patients with functional vertigo had significantly higher scores on the anxiety (HADS-A) and depression (HADS-D) subscales, while patients with organic vertigo had lower scores. This finding is consistent with our results, which showed that HADS-A and HADS-D had higher discriminatory power.^24^ Zhu et al (2020) investigated HADS scores in patients with vestibular migraine (and benign paroxysmal positional vertigo and found that patients with vestibular migraine had significantly higher HADS scores, while benign paroxysmal positional vertigo patients had lower scores. They proposed that HADS could help differentiate functional vertigo from organic causes, which is in line with our findings.^30^

The research by Russia et al (2023) focused on functional vertigo risk assessment for patients with benign paroxysmal positional vertigo. The study revealed that patients who experienced moderate anxiety and depression symptoms without showing any clinically important psychiatric conditions had risk of developing functional vertigo. The study established a link between anxiety and functional dizziness development, which supports our findings.^31^ Hashimoto et al (2021) found a positive correlation between HADS anxiety levels and functional vertigo episode frequency, with higher HADS-A scores associated with more frequent vertigo attacks. This further supports the link between anxiety and functional vertigo.^32^

### Clinical and Demographic Correlations

We found that functional vertigo patients were younger compared to peripheral vertigo patients (43.44 vs 60.14 years of age, *P* < .001). This finding suggests that functional vertigo should be considered as a differential diagnosis, especially in younger patients with dizziness. This age-related distinction is important, as it suggests that functional vertigo may often be overlooked in this demographic (as reported in prior research), which potentially could lead to misdiagnosis and inappropriate management.^14^ Prior research has also shown that a significant portion of patients with dizziness may have underlying mood or anxiety disorders. Studies by Nagaratnam et al and Ferrari et al reported similar findings, highlighting the need to consider psychosomatic factors when evaluating dizziness in younger patients, which is consistent with our findings.^33^,^34^ The study by Ventura et al showed that 52 of 189 patients with vertigo/dizziness complaints had medically unexplained symptoms. They found that non-organic vertigo patients exhibited psychogenic patterns more frequent than those with organic vertigo, which supports the argument that a significant portion of dizziness cases may arise from psychosomatic or functional factors.^35^

### Interplay with Anxiety and Depression

Our research demonstrates that anxiety and depression have complex relationships with vertigo. The identical VSS-SF-T scores between functional and peripheral vertigo groups indicate that symptom severity does not serve as a distinguishing characteristic. This raises an important question: Is anxiety and depression a reaction to vertigo symptoms, or a manifestation itself of underlying anxiety disorders? Although the VSS-SF-T scores were similar between the groups, a deeper analysis of the subscales revealed that VSS-SF-A scores were significantly higher in patients with functional vertigo, whereas VSS-SF-V scores were elevated in cases of peripheral vertigo. This suggests that mood disorders, such as anxiety and depression, may be associated with vertigo symptoms with similar severity even in the absence of organic vestibular pathology. These results support the hypothesis that functional vertigo originates from psychological factors rather than structural vestibular dysfunction, reinforcing the need for a multidimensional approach to the diagnosis and management of this often overlooked condition.

A review by Staab et al found that primary anxiety disorders can cause dizziness and that a thorough assessment is required to determine causality.^36^ Our findings support a strong association between psychological factors and functional dizziness, consistent with their hypothesis. Differentiating between symptom-induced anxiety and anxiety-induced vertigo is crucial for accurate diagnosis and proper treatment.

The observed strong correlations between HADS-A and VSS-SF-A (*r* = 0.695, *P* < .001) and HADS-D and VSS-SF-A (*r* = 0.609, *P* < .001) suggest an association between anxiety and depression and that they influence the severity of autonomic symptoms in functional vertigo patients. The results align with previous studies, which have shown that patients with functional vertigo commonly display increased autonomic dysfunction indicators associated with anxiety and depression.^1^,^37^ Our findings support previous studies by Staab and Lahmann et al (2015), which showed that functional vertigo produces greater autonomic dysfunction and anxiety symptoms than organic vestibular disorders. Lahmann discovered that patients with unexplained dizziness had higher psychiatric disorder rates, which supports the requirement for comprehensive diagnostic methods in patients without organic disease.^21^,^36^ Our research supports the expanding scientific evidence that shows functional vertigo results from impaired sensory integration and heightened autonomic reactions rather than actual vestibular system problems. The results demonstrate the necessity of combining psychological support with vestibular assessment in clinical practice.^36^

### Management Implications

The high correlation between HADS and VSS-SF-A scores indicates that anxiety and depression are associated with greater severity of vertigo symptoms in patients with functional dizziness, which supports the idea that functional vertigo patients should receive psychiatric treatment at an early stage.^28^ Research conducted by Limburg et al demonstrated that multimodal psychosomatic treatments effectively decreased dizziness severity in these patients, thus supporting the need for psychological support in functional vertigo management.^27^ Cognitive-behavioral therapy and pharmacological treatments, such as selective serotonin reuptake inhibitors, have been found to be useful in reducing both vertigo symptoms and associated psychological distress.^38^ The distinction between functional vertigo and peripheral vertigo is crucial, as misdiagnosis may lead to delays in appropriate psychological treatment and increased healthcare costs.^22^ Diagnosis of functional vertigo at an early stage is important to begin appropriate psychological treatments. Our study supports the use of psychometric tools in the ED to enhance diagnostic accuracy and early treatment planning for functional vertigo.

## LIMITATIONS

Our study has some limitations. The small sample size and single-center design limit generalizability, requiring validation with larger, multicenter studies. Our reliance on ENT assessments without formal psychiatric evaluation may limit the accuracy of functional vertigo diagnoses. Future studies should include psychiatric consultations to enhance diagnostic accuracy. Lack of follow-up data is another limitation; future research should assess long-term outcomes with repeated VSS and HADS scoring after treatment of the underlying physiological disorder.

A larger sample, multidisciplinary evaluation, and follow-up studies will enhance the reliability of scores to address functional vertigo diagnosis with the use of anxiety and depression scales in emergency settings. Another limitation lies in the performance of the clinical scales themselves. With sensitivities and specificities ranging from 68–88%, these tools are not diagnostic gold standards and are best used in conjunction with clinical judgement and other diagnostic criteria. The implementation of brief psychometric scales including VSS and HADS within ED and ENT settings could speed up the detection of functional vertigo. Early identification enables immediate psychological assessment and reduces the need for unnecessary diagnostic imaging and improves patient management. Future research should focus on creating standardized algorithms for functional vertigo triage and treatment to improve both patient care outcomes and efficiency.

Furthermore, the role of emergency physicians in diagnosing functional vertigo may be debated. While the primary responsibility in the ED is to rule out emergent conditions such as central vertigo, differentiating between peripheral and functional causes may exceed the typical scope of emergency care and require interdisciplinary collaboration. Future multicenter studies with larger, more diverse populations and inclusion of structured psychiatric evaluations are needed to enhance generalizability and reduce potential sources of bias. Research should investigate how early psychiatric interventions affect patient outcomes and healthcare costs

## CONCLUSION

Our research indicates that using the Vertigo Symptom Scale and Hospital Anxiety and Depression Scale in standard ED and ENT assessments show promise as supportive tools for identifying functional vertigo in emergency settings and may assist clinicians in the early recognition and management of this condition. These assessment tools have the potential to decrease the number of diagnostic procedures and reduce the time required for proper psychological treatment. Our study suggests that psychometric tools may aid in identifying functional vertigo in the ED setting, supporting the potential role of emergency clinicians in early diagnostic consideration and treatment planning.

## Figures and Tables

**Table 1:** Descriptive and baseline characteristics of patients diagnosed with functional vs peripheral vertigo in a study of dizziness-related emergency department visits.

Variable	Functional Vertigo (n = 25) Mean (SD), 95% CI	Peripheral Vertigo (n = 44) Mean (SD), 95% CI	P-value
Age (years)	43.44 (16.85), 36.49–50.39	60.14 (14.93), 55.60–64.68	<.001*
VSS-SF-V Score	6.56 (5.66), 4.22–8.90	13.05 (6.02), 11.22–14.87	<.001*
VSS-SF-A Score	9.04 (3.94), 7.41–10.67	3.80 (3.66), 2.68–4.91	<.001*
VSS-SF-T Score	15.60 (8.94),11.91–19.29	16.84 (7.39), 14.60–19.09	.54
HADS-A Score	9.28 (5.03), 7.20–11.36	4.18 (3.03),3.26–5.10	<.001*
HADS-D Score	7.52 (4.50), 5.66–9.38	2.91 (2.59), 2.12–3.70	<.001*
Sex, n (%)	n (%)	n (%)	.66
Female	15 (60.0)	24 (54.5)	—
Male	10 (40.0)	20 (45.5)	—

Student t-test was used for all continuous variables. Distribution by sex was analyzed via chi-square (χ^2^) test. P < .05 is considered statistically significant.

* P < .05 is considered statistically significant.

*VSS-SF-V*, Vertigo-balance subscale score; *VSS-SF-A*, Autonomic-anxiety subscale score; *VSS-SF-T*, Total vertigo symptom score; *HADS-A*, anxiety subscale of the Hospital Anxiety and Depression Scale; *HADS-D*, depression subscale of the Hospital Anxiety and Depression Scale.

**Table 2:** Correlation matrix among the scales used in a study of functional vertigo diagnosis in emergency settings.

Scale	VSS-SF-V r (P)	VSS-SF-A r (P)	VSS-SF-T r (P)	HADS-A r (P)	HADS-D r (P)
VSS-SF-V	1	−0.023 (.853)	0.823 (< .001) ^*^	−0.084 (.494)	−0.158 (.194)
VSS-SF-A	−0.023 (.853)	1	0.549 (<.001) ^*^	0.695 (< .001) ^*^	0.609 (< .001) ^*^
VSS-SF-T	0.823 (< .001) ^*^	0.549 (< .001) ^*^	1	0.325 (.006) ^*^	0.214 (.078)
HADS-A	−0.084 (.494)	0.695 (< .001) ^*^	0.325 (.006) ^*^	1	0.726 (< .001) ^*^
HADS-D	−0.158 (.194)	0.609 (< .001) ^*^	0.214 (.078)	0.726 (< .001) ^*^	1

Pearson correlation coefficients (r) are shown with P-values in parentheses. Correlations with P < .05 are marked with ^*^ to indicate statistical significance. Cases in which P < .001 are shown as < .001 for clarity.

*VSS-SF-V*, Vertigo-balance subscale score; *VSS-SF-A*, Autonomic-anxiety subscale score; *VSS-SF-T*, Total Vertigo Symptom Score; *HADS-A*, Anxiety subscale of the Hospital Anxiety and Depression Scale; *HADS-D*, Depression subscale of the Hospital Anxiety and Depression Scale.

**Table 3:** Diagnostic accuracy metrics for clinical scales used to differentiate functional and peripheral vertigo in a study of emergency department patients with dizziness.

**A**. Best diagnostic values for functional vertigo.								

Scale	Cut Point	Sensitivity (%)	Specificity (%)	PPV (%)	NPV (%)	Youden’s Index	AUC	Metric Score
HADS-A	8	68.0	88.6	77.3	83.0	0.566	0.807^*^	1.57
HADS-D	4	76.0	75.0	63.3	84.6	0.510	0.804^*^	1.51
VSS-sf-A	8	72.0	84.1	72.0	84.1	0.561	0.847^*^	1.56

**B**. Best diagnostic value for peripheral vertigo.								

Scale	Cut Point	Sensitivity (%)	Specificity (%)	PPV (%)	NPV (%)	Youden’s Index	AUC	Metric Score

VSS-sf-V	9	81.8	72.0	83.7	69.2	0.538	0.795^*^	1.54

Receiver operating characteristic (ROC) curve analyses were used to determine optimal cut points for diagnosing vertigo subtypes. AUC values marked with ^*^ denote statistical significance (P < .05)

*AUC*, area under the curve; *VSS-SF-V*, Vertigo-balance subscale score; *VSS-SF-A*, Autonomic-anxiety subscale score; *HADS-A*, Anxiety subscale of the Hospital Anxiety and Depression Scale; *HADS-D*, Depression subscale of the Hospital Anxiety and Depression Scale; *PPV*, positive predictive value; *NPV*, negative predictive value.

## References

[1] Brandt T, Huppert D, Strupp M (2015). Functional dizziness: diagnostic keys and differential diagnosis. J Neurol.

[2] Diukova GM, Zamergrad MV, Golubev VL (2017). Functional (psychogenic) vertigo. Zh Nevrol Psikhiatr Im S S Korsakova.

[3] Afzelius L, Henriksson NG, Wahlgren L (1980). Vertigo and dizziness of functional origin. Laryngoscope.

[4] Cao Z, Zhu C, Zhou Y (2021). Risk factors related balance disorder for patients with dizziness/vertigo. BMC Neurol.

[5] Garabet R, Tran D, Huang Z (2024). Ten year trends of dizziness in the emergency department.

[6] Kelly KM, Lima AA, Campbell TP, Campbell TP, Kelly KM (2023). Dizziness. Handbook of Emergency Neurology.

[7] De Waal MWM, Arnold IA, Eekhof JAH (2004). Somatoform disorders in general practice: prevalence, functional impairment and comorbidity with anxiety and depressive disorders. Br J Psychiatry.

[8] Eckhardt-Henn A, Breuer P, Thomalske C (2003). Anxiety disorders and other psychiatric subgroups in patients complaining of dizziness. J Anxiety Disord.

[9] Tusa RJ (2009). Dizziness. Med Clin North Am.

[10] Futami S, Miwa T (2024). Comprehensive equilibrium function tests for an accurate diagnosis in vertigo: a retrospective analysis. J Clin Med.

[11] Edlow JA (2019). The timing-and-triggers approach to the patient with acute dizziness. Emerg Med Pract.

[12] Edlow JA, Carpenter C, Akhter M (2023). Guidelines for Reasonable and Appropriate Care in the Emergency Department 3 (GRACE-3): acute dizziness and vertigo in the emergency department. Acad Emerg Med.

[13] Yanik B, Külcü DG, Kurtais Y (2008). The reliability and validity of the Vertigo Symptom Scale and the Vertigo Dizziness Imbalance questionnaires in a Turkish patient population with benign paroxysmal positional vertigo. J Vestib Res Equilib Orientat.

[14] Jang Y, Hur HJ, Park B (2024). Psychosocial factors associated with dizziness and chronic dizziness: a nationwide cross-sectional study. BMC Psychiatry.

[15] Tzartzas K, Aslan S, Kokkinakis I (2023). Managing a functional disorder with vertigo or dizziness in a primary care setting: clinical case. Eur Psychiatry.

[16] Spinhoven Ph, Ormel J, Sloekers PPA (1997). A validation study of the Hospital Anxiety and Depression Scale (HADS) in different groups of Dutch subjects. Psychol Med.

[17] Yardley L, Jahanshahi M, Hallam R, Bronstein AM, Brandt T, Woollacott M, Nutt JG (2004). Psychosocial aspects of disorders affecting balance and gait. Clinical Disorders of Balance, Posture and Gait.

[18] Aydemir O (1997). Validity and reliability of Turkish version of Hospital Anxiety and Depression Scale. Turk J Psychiatry.

[19] Bossuyt PM, Reitsma JB, Bruns DE (2015). STARD 2015: an updated list of essential items for reporting diagnostic accuracy studies. BMJ.

[20] Von Elm E, Altman DG, Egger M (2007). The Strengthening the Reporting of Observational Studies in Epidemiology (STROBE) Statement: guidelines for reporting observational studies*. Bull World Health Organ.

[21] Lahmann C, Henningsen P, Brandt T (2015). Psychiatric comorbidity and psychosocial impairment among patients with vertigo and dizziness. J Neurol Neurosurg Psych.

[22] Schmid G, Henningsen P, Dieterich M (2011). Psychotherapy in dizziness: a systematic review. J Neurol Neurosurg Psych.

[23] Bhattacharyya N, Gubbels SP, Schwartz SR (2017). Clinical practice guideline: benign paroxysmal positional vertigo (update). O. tolaryngol Neck Surg.

[24] Tschan R, Wiltink J, Best C (2008). Validation of the German version of the Vertigo Symptom Scale (VSS) in patients with organic or somatoform dizziness and healthy controls. J Neurol.

[25] El Abedein AMZ, Talaat HS, Gad ME (2020). Development of the Arabic version of the Vertigo Symptom Scale-Short Form: validity and reliability. Menoufia Med J.

[26] Dyukova GM, Kryukov AI, Makarov SA (2021). A method for prediction functional dizziness after benign paroxysmal positional vertigo. Zh Nevrol Psikhiatr Im S S Korsakova.

[27] Limburg K, Schmid-Mühlbauer G, Sattel H (2019). Potential effects of multimodal psychosomatic inpatient treatment for patients with functional vertigo and dizziness symptoms – A pilot trial. Psychol Psychother Theory Res Pract.

[28] Godemann F, Schabowska A, Naetebusch B (2006). The impact of cognitions on the development of panic and somatoform disorders: a prospective study in patients with vestibular neuritis. Psychol Med.

[29] Piker EG, Kaylie DM, Garrison D (2015). Hospital Anxiety and Depression Scale: factor structure, internal consistency and convergent validity in patients with dizziness. Audiol Neurotol.

[30] Zhu C, Li Y, Ju Y (2020). Dizziness handicap and anxiety depression among patients with benign paroxysmal positional vertigo and vestibular migraine. Medicine (Baltimore).

[31] Elovikov AM, Russia AWPSMU Perm, 614000, Borodulina II, Perm Regional Clinical Hospital, Perm, 614990, Russia, Karakulova YuV, Academician Wagner Perm State Medical University, Perm, 614000, Russia (2023). Symptoms of anxiety and depression in patients with benign paroxysmal positional vertigo. Russ Otorhinolaryngol.

[32] Hashimoto K, Hashizume M (2021). Relationship between somatosensory amplification and frequency of vertigo episodes: a study of psychogenic vertigo. Equilib Res.

[33] Ferrari S, Monzani D, Baraldi S (2014). Vertigo “in the pink”: the impact of female gender on psychiatric-psychosomatic comorbidity in benign paroxysmal positional vertigo patients. Psychosomatics.

[34] Nagaratnam N, Ip J, Bou-Haidar P (2005). The vestibular dysfunction and anxiety disorder interface: a descriptive study with special reference to the elderly. Arch Gerontol Geriatr.

[35] Ventura J, Liberman RP, Green MF (1998). Training and quality assurance with the structured clinical interview for DSM-IV (SCID-I/P). Psychiatry Res.

[36] Staab JP, Ruckenstein MJ (2003). Which comes first? Psychogenic dizziness versus otogenic anxiety. Laryngoscope.

[37] Eckhardt-Henn A, Best C, Bense S (2008). Psychiatric comorbidity in different organic vertigo syndromes. J Neurol.

[38] Wang A, Fleischman KM (2021). Persistent postural-perceptual dizziness in children and adolescents. Otol Neurotol.

